# Prognostic Significance of Fms-Like Tyrosine Kinase 3 Internal Tandem Duplication Mutation in Non-Transplant Adult Patients with Acute Myeloblastic Leukemia: A Systematic Review and Meta-Analysis

**DOI:** 10.31557/APJCP.2020.21.10.2827

**Published:** 2020-10

**Authors:** Ikhwan Rinaldi, Melva Louisa, Fikri Ichsan Wiguna, Elizabeth Budiani, Jeffrey Christian Mahardhika, Khairul Hukmi

**Affiliations:** 1 *Department of Internal Medicine Division of Hematology and Medical Oncology, Faculty of Medicine Universitas Indonesia-Dr. Cipto Mangunkusumo National General Hospital, Jakarta, Indonesia. *; 2 *Department of Pharmacology and Therapeutics, Faculty of Medicine, Universitas Indonesia, Indonesia. *; 3 *Faculty of Medicine, Universitas Indonesia, Indonesia. *

**Keywords:** Leukemia, myeloid, acute, Fms-like tyrosine kinase 3, prognostic significance

## Abstract

**Background:**

Fms-like tyrosine kinase-3, internal tandem duplication (FLT3-ITD) mutation, is a known predictor for worse outcome in patients with acute myeloblastic leukemia (AML). However, the prognostic significance of FLT3-ITD mutation in adult, non-transplant patients is still unclear therefore we conducted a systematic review and meta-analysis to explain this issue. The main outcome was overall survival (OS), while additional outcomes included event-free survival (EFS).

**Methods:**

Seven Databases (ScienceDirect, Scopus, PubMed, Cochrane, SpringerLink, ProQuest, and EBSCOhost) were searched up to August 2020. Studies investigating the prognostic value of AML in adults with FLT3-ITD mutational status were selected. Studies which patients had received transplantation, diagnosed with acute promyelocytic leukemia (APL) or secondary AML were excluded. The selected studies were divided into subgroups based on their cytogenetic profile. Summary hazard ratios (HR) and 95% confidence intervals (CI) were calculated using fixed-effects models. Heterogeneity tests were conducted and presented in I_2_ value. Forest plot was presented to facilitate understanding of the results. Publication bias was analyzed by Funnel Plot test.

**Results:**

A total of ten studies describing research conducted from 1999 to 2020, met the inclusion criteria for this study. Nine studies reported OS and four studies reported EFS in HR. The highest HR for OS is 6.33 (95% CI, 2.61-15.33; p < 0.001), for EFS is 3.58 (95% CI, 1.59 – 8.05); p = 0.002)., while the lowest for OS is 1.33 (95% CI, 0.88-2.01; P = 0.174) and for EFS is 1.29 (95% CI, 0.75-2.23; p = 0.34). Nine studies were included in meta-analysis with HR for OS 1.91 (95% CI, 1.59–2.30, p < 0.00001), whereas 4 studies were included in meta-analysis for EFS with HR 1.64 (95% CI, 1.25–2.14; p = 0.0003).

**Conclusion:**

FLT3-ITD mutation is associated with worse prognosis in adult, non-transplant patients with AML, both for OS and EFS.

## Introduction

Acute myeloblastic leukemia is a hematological malignancy with clonal expansion of myeloid blast in bone marrow and peripheral blood. This type of leukemia most commonly occurs in adults, accounting for 50% of adult leukemia, while it represents only 15% to 20% of childhood leukemia (Nasir et al., 2016). In 2012, the incidence of AML worldwide reached 351,965 cases, and the age-standardized rate increased up to 4.7/100,000 lives with a 5-year prevalence of approximately 1.5% (Union for International Cancer Control, 2014).

This type of leukemia is a heterogeneous disease; many factors influence its prognosis, such as age, clinical manifestation at the time of diagnosis, somatic mutation, and cytogenetic abnormality One factor that has meaningful prognostic effect is mutation of FLT3 (De Kouchkovsky and Abdul-Hay, 2016). The most common type of FLT3 mutation is ITD in the juxtamembrane domain (Whitman et al., 2001). The genetic mutation of a patient with AML was considered to be lower in pediatric patients (0–14 years old) than in adult patients (≥15 years of age) (Dang et al., 2013). There was a 4% prevalence of FLT3-ITD in pediatric patients with AML compared to a 26% prevalence in adult patients with AML (Juhl-christensen et al., 2012).

The therapeutic option for patients with AML was chosen based on the disease severity and phase. Allogenic hematopoietic stem cell transplantation (HSCT) has become the therapeutic option for patients with AML, particularly those with the severe form of AML (Fenaux et al., 2014). However, HSCT has become an economic burden because of its high cost (Gratwohl, 2010). HSCT is related to gross national income per capita and government healthcare expenditure (Gratwohl, 2010) The activity of HSCT is concentrated in a region that has higher governmental healthcare expenditures and higher gross national income per capita (Gratwohl, 2010). Moreover, no HSCT was performed in a region or a country with a gross national income per capita of less than $700 (USD) (Gratwohl, 2010).

Several earlier studies revealed that FLT3-ITD mutation has a worse prognostic value than the wild-type and several other mutations (Whitman et al., 2001; Ponziani et al., 2006; Shamaa et al., 2014; Sharawat et al., 2016; Scholl et al., 2008; Dang et al., 2013). Moreover, a systematic review and a meta-analysis about the prognostic significance of FLT3-ITD mutation in patients with AML reported that FLT3-ITD positivity significantly and adversely affected the prognosis of patients with AML (Port et al., 2014).

Yet, there is still no review that discusses prognostic significance specifically in non-transplanted patients. We aim to focus on this specific population because transplantation might not always be performed due to its economic burden. Thus, the prognosis of patients with that condition is still unclear. We conducted this systematic review to enhance our understanding and determine optimal treatment for this kind of population. In our study, we only included adult patients because AML and FLT3-ITD are both more common in adults than in younger patients.

## Materials and Methods

Our review, “Prognostic significance of FLT3 internal tandem duplication mutation in non-transplant adult patients with AML: a systematic review and meta-analysis” (ID: CRD42019139349), has been registered to PROSPERO (International prospective register of systematic reviews) by the National Institute for Health Research (NIHR).

*Search strategy*

For this review, a systematic literature search was conducted in seven databases: ScienceDirect, Scopus, PubMed, Cochrane, SpringerLink, ProQuest, and EBSCOhost up to August 2020. We searched for studies that were performed in the adult AML population, that reported FLT3-ITD mutation status, and described outcome with regard to prognosis or survival. The keywords used were: ((acute myeloid leukemia) OR (acute myelocytic leukemia) OR (acute myelogenous leukemia) OR (AML)) AND (FLT3) AND ((internal tandem duplication) OR (ITD)) AND ((prognosis) OR (prognostic) OR (survival)) NOT (pediatric). There was no language limitation in this search. Studies that were written in language other than English were translated using an online translator engine. Hand searching was not conducted. Full-text articles were obtained using our institutional access to databases. 

*Inclusion and exclusion criteria*

We included studies that had sample patients who had been diagnosed with AML, had FLT3-ITD status (whether positive or negative), ranged in age ≥15 years old, and had prognostic value at least in overall survival (OS). Studies that discussed other mutations were included as long as OS data based on FLT3-ITD status were available. Studies that contained normal and abnormal karyotype were included. We excluded studies which involved subjects who had received transplantation, were diagnosed with acute promyelocytic leukemia (APL), and had AML that develops from another disease or condition (e.g., myelodysplastic syndrome and drug-induced AML) or commonly called secondary AML. In this systematic literature search, we did not include experimental studies conducted in animals.

*Data extraction*

Literature search was conducted according to Preferred Reporting Items for Systematic Reviews and Meta-Analyses (PRISMA) 2009. We identified studies in databases, removed duplicates, screened for inclusion criteria, and assessed eligibility. Eligibility was assessed using a “tool to assess risk of bias in cohort studies” from Cochrane, because all of the studies that were ready for assessment were cohort studies. The assessment results were compiled using Review Manager 5.3.

Literature search, screening, and eligibility assessment were performed by three reviewers. Screening for titles and abstracts was done independently, based on aforementioned inclusion criteria. Furthermore, each study was assessed for eligibility by two reviewers independently. Disagreements between reviewers were resolved by discussion with the third reviewer. All of the reviewers extracted data from all of the studies, such as author, year of publication, design, sample size, population, sample ages, number of sample with FLT3-ITD mutation, and prognostic outcome such as OS, EFS, disease-free survival (DFS), relapse-free survival (RFS) and complete remission (CR) value (e.g., using hazard ratio, median, or rate).

The data were analyzed statistically and meta-analysis was performed using Review Manager 5.3. There might be slight differences between original numbers in study and table results from meta-analysis, because the original numbers had to be converted into Review Manager 5.3 format. Studies were analyzed in subgroups based on whether the studies included only normal karyotype population or diverse karyotype (both normal and abnormal karyotype were included as population). Hazard ratio was calculated using an inverse variance method with fixed-effects model to represent primary and secondary outcomes. The results were presented with total values, 95% CI, and I_2_ to assess heterogeneity. Low, moderate and high heterogeneity associated to I_2_ values of 25%, 50% and 75% with I_2_ value higher than 50% considered having significant heterogeneity (Sedgwick, 2015). Forest plot was used to facilitate understanding the results. Funnel plot was presented to demonstrate the plausibility of publication bias in this review.

## Results

*Study characteristics*

**Figure 1:**
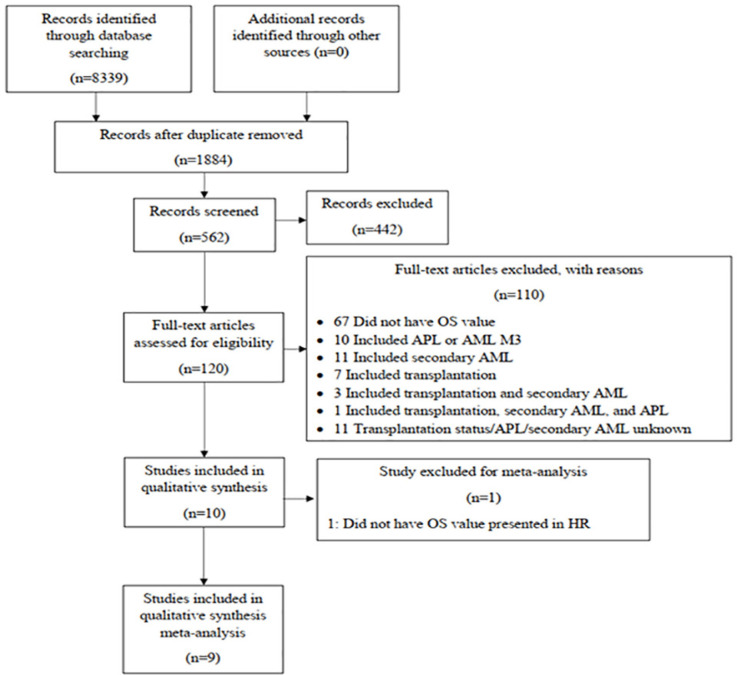
PRISMA Flow Chart for Searching Strategy

We assessed a total of 106 full-text articles to determine eligibility for this study. We contacted all corresponding authors of 11 studies that did not mention transplantation, APL, or secondary AML status. However, we only received one response, and we were unable to obtain the data due to protocol rules from the original sample database. Thus, all of those studies were excluded from this review. As shown in Figure 1 and Table 1, ten studies met the inclusion criteria, all of which were published from 1999 to 2020. A total of 1,513 subjects with age ranges from 15 years old to 91 years old were included in our systematic review. Every study has a different therapeutic regimen for its participants. All studies only included adult patients with median age ranges from 42 years old to 54 years old. However, in 3 studies from Dang et al., (2013), Fujiwara et al., (2019), and Ni et al., (2020), only the elderly population (≥ 60 years old), with median age ranges from 67 years old to 71 years old, matched with inclusion criteria. The number of mutated FLT3-ITD patients in every study varied, ranging from 5.67% to 25.93% of population, which was sufficient to be analyzed. The lowest FLT3-ITD mutation frequency was shown in the study by Niparuck (2019), while the highest was in Preudhomme et al., (2002). 

*Risk of bias*

**Figure 2:**
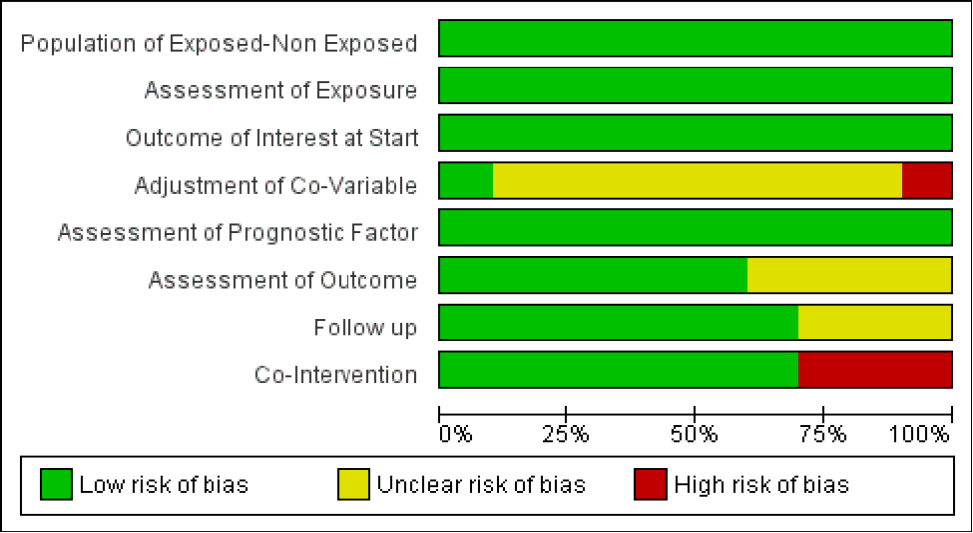
Summary of Risk of Bias Analysis among Studies

**Figure 3:**
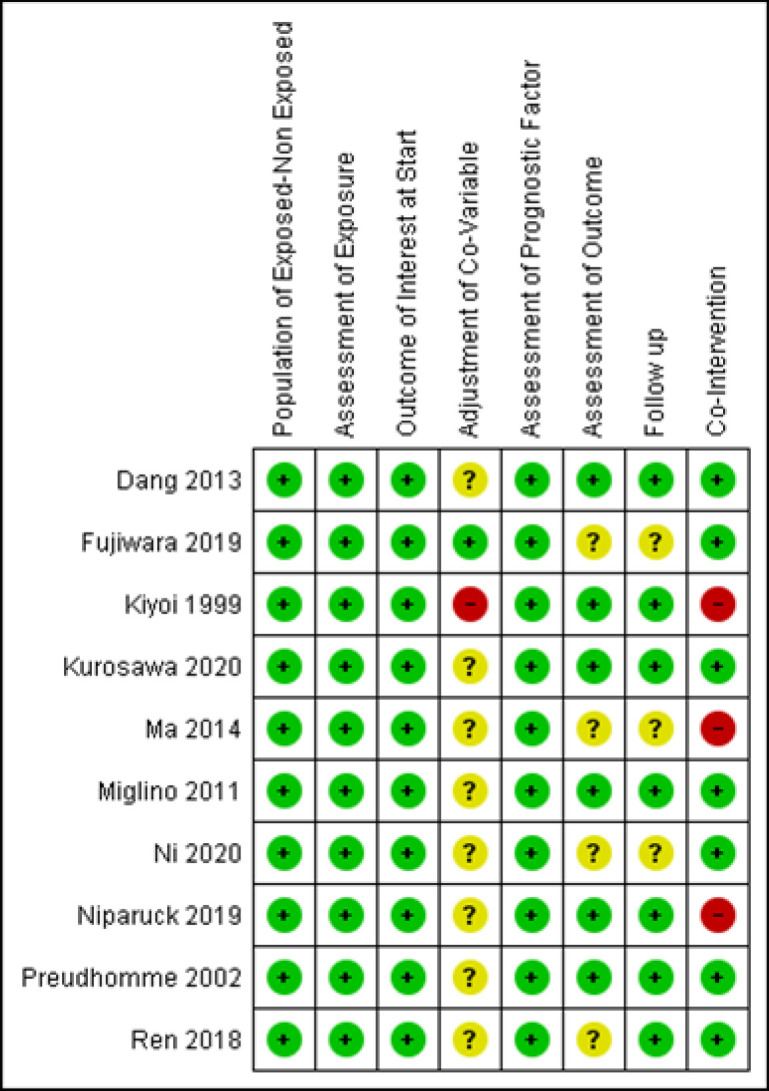
Intra-Study Analysis of Risk of Bias

All of the studies had low risk of bias in selecting the population for both FLT3-ITD mutation and wild-type groups, assessment of exposure, assessment of prognostic factor, and outcome of interest at the start (Figure 2). As for the following process, nearly all of the studies had low risk of bias, which meant no missing outcome data (Figure 2). Ma et al., (2015) had unclear risk of bias for following-up because the study did not include all subjects in the analysis and did not provide supporting reasons (Figure 3). The follow-up duration was relatively short in Ni et al., (2019). All studies did not have blinding or double-blinding for the assessment of outcomes (OS, EFS, and complete remission) (Figure 2). However, the outcomes were, for the most part, drawn from medical records and, therefore, very unlikely to introduce biases. Four studies had unclear risk of bias. Ma et al., (2015) and Ni et al., (2020) did not state how they reached the outcomes clearly, Ren et al., (2018) using phone-based follow-up to obtain the outcomes did not mention whether they did any blinding, while the study from Fujiwara et al., (2019) did not describe how the follow up was conducted. Kiyoi et al., (1999), Ma et al., (2015), and Niparuck et al., (2019) did not have the similar co-intervention between the exposed and the non-exposed groups; thus, all of them had high risk of bias regarding co-intervention (Figure 3). The majority of studies did not mention whether there was matching of co-variables between groups (Figure 1), nor was there explanation of the baseline characteristics between FLT3-ITD mutation group and FLT3-ITD wild-type group. Most of studies did not mention overall survival value based on cytogenetic status in wild-type or mutant groups. Thus, they were considered to have unclear risk of bias. The study by Kiyoi et al., (1999) had high risk of bias due to significant differences of age and white blood count between groups. The study from Kurosawa et al., (2020) stated that there was a possibility of selection bias since the study used a retrospective design.

Funnel plot test of 9 studies included in the meta-analysis, using hazard ratios showed that 8 studies were distributed symmetrically, indicating that publication bias in this meta-analysis was little to none (Figure 6). Only one study from Dang, et al., (2013) had outliers. 

*Prognostic Outcome*

We extracted outcomes in these studies which varied from HR for OS, median OS, OS rate, HR of EFS, HR for DFS/RFS, EFS rate, DFS/RFS rate, and odds ratio (OR) for CR. The studies were classified into two subgroups, normal and diverse karyotype. All of the studies but one by Niparuck et al., (2019) reported HR for OS. The study by Dang et al., (2013) reported the highest HR for OS (HR, 6.33; 95% CI, 2.61–15.33; p < 0.001). The lowest HR for OS was reported by Ma et al., (2015) (HR, 1.33; 95% CI, 0.88–2.01; p = 0.174). At 12 months, the overall survival rate in mutated patients had already started to decrease compared to the wild-type population.

Four studies reported HR for EFS. Dang (2013) reported the highest (HR, 3.58; 95% CI, 1.59 – 8.05); p = 0.002) while Miglino et al., (2011) reported the lowest (HR, 1.29; 95% CI, 0.75–2.23; p = 0.34). More results from all of the included studies are shown in Table 1.

**Figure 4:**
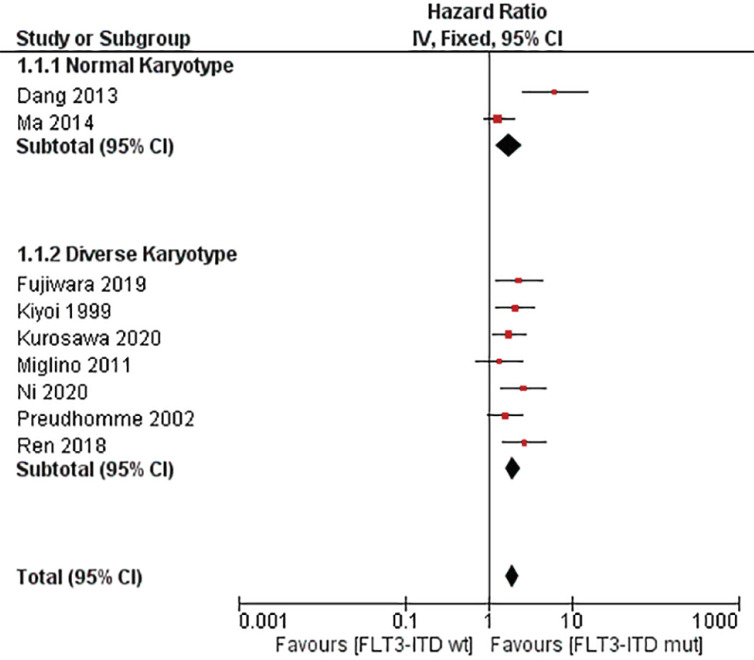
Forest Plot of the Association between FLT3-ITD Mutation and Overall Survival (OS) among Adult Acute Myeloblastic Leukemia Ppatients

The values of HR with 95% CI for OS were extracted from the studies mentioned, with the exception of the study by Niparuck et al., (2019). Fixed effect models were employed. This analysis of nine studies showed that overall HR for OS is 1.91 (95% CI, 1.59–2.30, p = < 0.00001) with I^2^ = 46%. We attempted subgroup analysis which yielded subtotal HR 1.76 (95% CI, 1.21-2.56, p = 0.003) with I^2^ = 90% and HR 1.96 (95% CI, 1.59-2.42, p = <0.00001) with I^2^ = 0% for normal and diverse karyotype group, respectively. Test for subgroup differences indicate homogeneity with I^2^ = 0%. (Figure 4 and Table 2)

**Figure 5:**
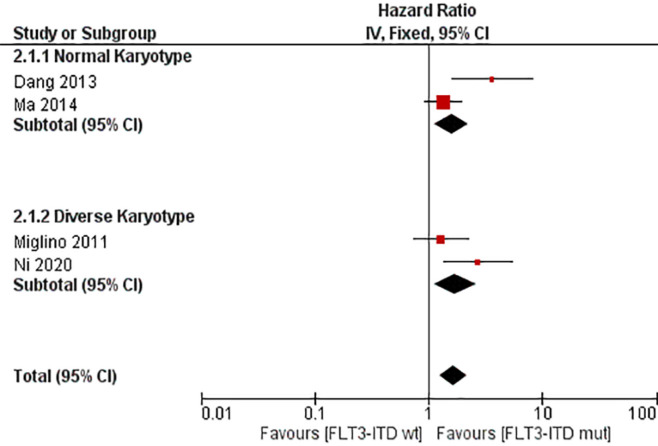
Forest Plot of the Association between FLT3-ITD Mutation and Event-Free Survival (EFS) among Adult, Acute Myeloblastic Leukemia Patient

Analysis for secondary outcome to determine the association between FLT3-ITD mutation and EFS was performed, including four studies that reported HR value for EFS. Mutation of FLT3-ITD was found to be associated with unfavorable prognosis as EFS value in HR 1.64 (95% CI, 1.26–2.14; p = 0.0003). However, the heterogeneity was significantly high with I^2^ = 60%. We conducted subgroup analysis for normal and diverse karyotype which yielded HR 1.59 (95% CI, 1.13-2.25; p = 0.008) with I^2^ = 79% and HR 1.71 (95% CI, 1.12-2.62; p = 0.01) with I^2^ = 64%. Test for subgroup differences yielded I^2^ = 0%, which indicated homogeneity. (Figure 5 and Table 3)

**Table 1:** List of Studies Included in the Systematic Review and Ooutcomes

Author	Year	Design	Size	Population	Age	Number of FLT3-ITD (+) (%)	HR OS	HR EFS/DFS/RFS (95% CI)	OS FLT3-ITD mutation positive vs negative	EFS/DFS/RFS FLT-ITD mutation positive vs negative	OR CR (95% CI)
					(years)		(95% CI)				
Dang et al.[6]	2013	Cohort	144 subjects (but only 76 had FLT3-ITD status)	Elderly patients (>60 years) diagnosed with de novo AML and normal karyotype	Median 71 (60–89)	13 (17.11)	6.33 (2.61–15.33); p ≤ 0.001	3.58 (1.59–8.05); p = 0.002 (EFS)	Not available	Not available	Not available
Fujiwara et al. [9]	2019	Cohort	281 subjects, but only 98 elderly subjects (≥65 years old) included in survival analysis)	Patients with de novo AML, non-M3-FAB type AML, and not therapy-related AML without previous hematological disease or family history of myeloid neoplasms	Median age of elderly subjects 71 (65-91)	20 (20,41)	2.316 (1.216 – 4.411); p = 0.011	14.603 (1.755 – 121.528); p = 0.013 (RFS)	Not available	Not available	Not available
Kiyoi et al.[13]	1999	Cohort	201 subjects	Adult patients diagnosed with de novo AML	Median 49 (not available)	46 (22.9)	2.10 (1.20–3.40); p ≤ 0.001	Not available	14.0% vs 44.6%; p≤0.001 (60 months)	20.0% vs 53.9 %; p = 0.001 (DFS at 60 months)	Not available
Kurosawa et al. [14]	2020	Cohort	235 subjects, but only 152 subjects who relapsed after CR1 had Hazard Ratio of FLT3-ITD mutation)	Adult patients (18-65) with AML, diagnosed between 1999-2010 with intermediate- or unknown risk AML according to Southwest Oncology Group (SWOG) cytogenetic classification and who had achieved CR1 with one or two courses of chemotherapy.	Median 51 (18-65)	38 (25)	1.75 (1.11 – 2.77); p = 0.017	Not available	19.0% vs 41.0%; p = 0.002 (2 years after relapse)	6% vs 26% (RFS at 2 years after relapse)	Not available
Ma et al.[16]	2014	Cohort	320 subjects	Patients ranged from 16 to 85 years old, diagnosed with de novo AML, and normal karyotype	Median 49 (16–85)	51 (15.94)	1.33 (0.88–2.01); p = 0.174	1.34 (0.92–1.96); p = 0.124 (EFS)	Not available	Not available	0.43 (0.22-0.85); p=0.015
Miglino et al.[17]	2011	Cohort	100 subjects	Patients diagnosed with de novo AML, non-M3 with intermediate and unfavorable karyotype	Median 46 (on 55 subjects <60 years old), median 69 (on 45 subjects ≥60 years old)	21 (21.00)	1.35 (0.71–2.59); p = 0.35	1.29 (0.75–2.23); p = 0.34 (EFS)	20.0% vs 38.0%; p = 0.03 (30 months)	6% vs 30%; p=0.01 (EFS at 30 months)	Not available
Ni et al. [19]	2020	Cohort	208 subjects, but only 92 elderly subjects aged 60-75 years old who did not have transplantation procedure)	Patients with de novo AML diagnosed between December 2016 and December 2019, non-M3-FAB type AML.	Median age of elderly subjects 67 (60-75)	17 (18.48)	2.662 (1.287 – 4.530); p = 0.038	2.689 (1.138 – 4.490); p = 0.034 (EFS)	Not available	Not available	Not available
Niparuck et al.[21]	2019	Cohort	141 subjects	Patients >15 years old diagnosed with de novo AML	Median 54 (15–88)	8 (5.67)	not available	Not available	25.0% vs 39.0%; p = 0.047 (12 months)	20% vs 41%; p=0.047 (RFS at 12 months)	Not available
Preudhomme et al.[27]	2002	Cohort	135 subjects	Patients ranged from 15 to 65 years old, diagnosed with de novo AML, and non-M3-FAB type AML	Median 45 (15–64)	35 (25.93)	1.58 (0.96–2.58)	Not available	14.0% vs 29.0%; p = 0.11 (60 months)	Not available	Not available
Ren et al.[28]	2018	Cohort	198 subjects	Adult patients with de novo AML	Median 42 (18–62)	28 (14.14)	2.70 (1.50–4.80); p = 0.001	2.8 (1.7-4.8); p ≤ 0.001 (DFS)	27.8% vs 67.2%; p=0.004	11.8% vs 52.5%; p≤0.001 (DFS)	Not available

FLT3, fms-like tyrosine kinase 3; ITD, internal tandem duplication; AML, acute myeloblastic leukemia; FAB, French-American-British; CR1, First Complete Remission; SWOG, Southwest Oncology Group, HR, hazard ratio; OS, overall survival; OR, odds ratio EFS, event free survival; DFS, disease free survival; RFS, relapse free survival; vs, versus

**Table 2:** Meta-Analysis of the Association between FLT3-ITD Mutation and Overall Survival (OS) among Adult, Acute Myeloblastic Leukemia

Study or Subgroup	log [Hazard Ratio]	SE	Weight	Hazard Ratio IV, Fixed, 95% CI
1.1.1 Normal Karyotype				
Dang 2013	1.845	0.452	4.3%	6.33 [2.61,15.35]
Ma 2014	0.285	0.211	19.5%	1.33 [0.88, 2.01]
Subtotal (95% CI)			23.8%	1.76 [1.21, 2.56]
Heterogeneity: Chi^2^ = 9.78, df = 1 (P = 0.002); I^2^ = 90%				
Test for overall effect: Z = 2.95 (P = 0.003)				
1.1.2 Diverse Karyotype				
Fujiwara 2019	0.84	0.329	8.0%	2.32 [1.22, 4.41]
Kiyoi 1999	0.742	0.266	12.3%	2.10 [1.25, 3.54]
Kurosawa 2020	0.56	0.233	16.0%	1.75 [1.11, 2.76]
Miglino 2011	0.3	0.33	8.0%	1.35 [0.71, 2.58]
Ni 2020	0.979	0.321	8.4%	1.66 [1.42, 4.99]
Preudhomme 2002	0.457	0.252	13.7%	1.58 [0.96, 2.59]
Ren 2018	0.993	0.297	9.8%	2.70 [1.51, 4.83]
Subtotal (95% CI)			76.2%	1.96 [1.59, 2.42]
Heterogeneity: Ch^i2^ = 4.64, df = 6 (P = 0.59); I^2^ = 0%				
Test for overall effect: Z = 6.33 (P < 0.00001)				
Total (95% CI)			100.0%	1.91 [1.59, 2.30]
Heterogeneity: Chi^2^ = 14.68, df = 8 (P = 0.07); I^2^ = 46%				
Test for overall effect: Z = 6.96 (P < 0.00001)				
Test for subgroup differences: Chi^2^ = 0.26, df = 1 (P = 0.61), I^2^ = 0%				

FLT3, fms-like tyrosine kinase 3; ITD, internal tandem duplication; OS, overall survival; CI, confidence interval

**Table 3:** Meta-Analysis of the Association between FLT3-ITD Mutation and Event-Free Survival (EFS) among Adult, Acute Myeloblastic Leukemia Patients

Study or Subgroup	log [Hazard Ratio]	SE	Weight	Hazard Ratio IV, Fixed, 95% CI
1.1.1 Normal Karyotype				
Dang 2013	1.28	0.414	10.8%	3.60 [1.60, 8.10]
Ma 2014	0.29	0.193	49.9%	1.34 [0.92, 1.95]
Subtotal (95% CI)			60.8%	1.59 [1.13, 2.25]
Heterogeneity: Chi^2^ = 4.70, df = 1 (P = 0.03); I^2^ = 79%				
Test for overall effect: Z = 2.67 (P = 0.008)				
1.1.2 Diverse Karyotype				
Miglino 2011	0.25	0.278	24.1%	1.28 [0.74, 2.21]
Ni 2020	0.99	0.35	15.2%	1.69 [1.36, 5.34]
Subtotal (95% CI)			39.2%	1.71 [1.12, 2.62]
Heterogeneity: Chi^2^ = 2.74, df = 1 (P = 0.10); I^2^ = 64%				
Test for overall effect: Z = 2.46 (P < 0.01)				
Total (95% CI)			100.0%	1.64 [1.25, 2.14]
Heterogeneity: Chi^2^ = 7.50, df = 3 (P = 0.06); I^2^ = 60%				
Test for overall effect: Z = 3.62 (P < 0.0003)				
Test for subgroup differences: Chi^2^ = 0.06, df = 1 (P = 0.80), I^2^ = 0%				

FLT3, fms-like tyrosine kinase 3; ITD, internal tandem duplication; EFS, event free survivalival; CI, confidence interval

**Figure 6:**
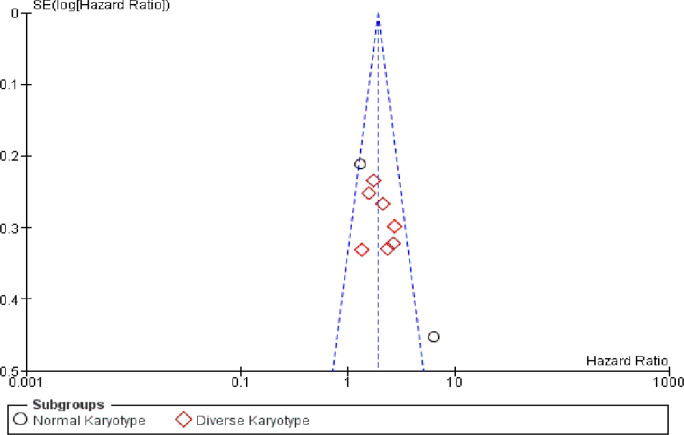
Funnel Plot of the Association between FLT3-ITD Mutation and Overall Survival (OS) among Adult Acute Myeloblastic Leukemia Patients

## Discussion

Genetic mutations are important prognostic factors in AML. One of the most well-known gene mutations in patients with AML is FLT3-ITD mutation (Whitman et al., 2001). Many studies have reported that this mutation is related to worse outcome in patients with AML (Port et al., 2014). However, it is important to know the prognostic significance of FLT3-ITD mutation in specific populations, such as patients who did not receive stem cell transplantation as a part of their therapy. We included studies of only patient populations who did not receive transplant therapy. Despite its efficacy in curing patients with AML, transplant therapy is always accompanied by economic burden (Gratwohl, 2010). In this study, we excluded patients with APL and secondary AML. Patients with APL were excluded due to different prognoses and treatment protocols (Coombs et al., 2015). Patients with secondary AML were excluded because this group of patients typically have a poorer outcome compared to patients with de novo AML, which could interfere with the outcome results related to FLT3-ITD mutation. Several factors contributing to poorer outcome in patients with secondary AML are older age at the time of diagnosis, more comorbidities and organ dysfunction, existence of persistent malignant disease, and prolonged myelosuppression due to prior treatment of myelodysplastic syndrome (Østgård et al., 2015; Boddu et al., 2017).

From the assessment of biases in Figure 2; Figure 3, it is implied that the overall quality of the studies included in this systematic review are quite good; thus, the data included have high reliability. The study by Kiyoi et al., (1999) had high risk of biases for adjustment of co-variables and co-intervention, which could disturb the outcome. The studies by Niparuck et al., (2019) and Ma et al., (2015) also had high risk of bias for co-intervention, thus downgrading the quality of the study. In addition, the study by Ma et al., (2015) showed questionable risk of biases in adjustment of co-variables, assessment of outcomes, and follow-up (Figure 3). For studies that had unclear risk of biases, we contacted the corresponding authors of the studies, and we received only one response. However, we still could not obtain the data due to protocol rules from the original sample databases.

From our funnel plot graphs (Figure 6) we could imply that the plausibility of publication bias in this study was quite little. Eight studies were distributed in the top of the graphs, indicating low standard errors of the studies. Dang et al., (2013) was distributed in the right bottom side of the graph, which means that it had larger standard errors (Sterne et al., 2004). This phenomenon could be explained by its small sample size. Dang et al., (2013) only analyzed 76 out of 144 samples which could intensify the outcome from what it should be.

All studies showed that mutated FLT3-ITD patients will have worse prognosis regarding hazard ratio of OS or EFS, as shown in Table 1. While OS was quite similar across studies, there was variation among the studies regarding the EFS rate. Miglino et al., (2011) showed that EFS rate was much lower than that shown in the other study probably because the study included only patients with intermediate and unfavorable risk karyotype, based on karyotypic subgroups defined by Grimwade et al., (2010). Miglino et al., (2011) included 81 subjects with intermediate-risk karyotypes (77 had normal karyotype, one patient had -Y, two had +8, one had +22) and 19 subjects with unfavorable karyotypes (15 had complex karyotype, three had monosomy 7, one had monosomy 7 and +22). Lin et al., (2017) showed that unfavorable karyotype would indicate much lower survival rate than other karyotype groups, and that the intermediate group who had mutations such as FLT3-ITD would also demonstrate an unfavorable outcome.

We could not include all studies in meta-analysis. A study by Niparuck et al., (2019) did not calculate HR values of OS in patients with FLT3-ITD mutation and only four studies analyzed EFS in the form of HR. Therefore, their results could not be pooled with data from other studies.

Based on our meta-analysis, FLT3-ITD mutation worsens the OS in non-transplant AML patients. Even though all studies favor negatively impact OS value, the results were heterogeneous. This might be caused by different populations regarding cytogenetic profile. Normal karyotype population tends to have diverse clinical expression, thus making heterogeneity value considered high. Walker et al., (2012) and Nimer et al., (2008) described that AML with normal karyotype has diverse clinical expression and led to wide range of survival rate. As shown in Figure 4 and Table 2, after dividing into 2 subgroups, studies in diverse karyotype was considered homogenous, whereas normal karyotype have high heterogeneity. Dang et al., (2013) has higher HR value. Besides its normal karyotype, Dang et al., (2013) only included elderly population which tends to has more comorbidity, frailty and biological differences, compared with younger patients (Almeida and Ramos, 2016). Study by Oran et al., (2012) demonstrated that comorbidities progressively worsened in older patients and were associated with less aggressive leukemia therapy and more early deaths. Poor prognosis for older patients of AML due to their cytogenetics profile was also implied in the study by Prassek et al., (2018). Analysis between subgroup showed that both favors FLT3-ITD positive would negatively impact OS value. 

The results correspond to another meta-analysis in different studies that analyzed similar populations but included patients who received stem cell transplantation. They showed HR values of OS as 1.86 (95% CI, 1.57–2.20) (Port et al., 2014) and 1.68 (95% CI, 1.39–2.03) (Yanada et al., 2005). Another study that only included patients who received allogeneic stem cell transplantation yielded similar results, with HR values of 1.94 (95% CI, 1.03–3.65) (Ardestani et al., 2018) and 2.98 (95% CI, 1.087–8.177) (Tang et al., 2017). These results imply that FLT3-ITD mutation in populations that did not receive transplantation carries similar risk as the transplanted population in terms of OS.

Event-free survival also counted in the meta-analysis from four studies, as shown in Figure 5 and Table 3. It showed that EFS in FLT3-ITD positive non-transplant population is worse than the wild-type population. This data has high heterogeneity, but from subgroup analysis we can see that the heterogeneity was not caused by cytogenetic profile difference. This can be concluded as in both subgroup, either normal or diverse karyotype, the heterogeneities were all high. Other factors might be contributed, and future research is needed. Besides, both subgroups favor FLT3-ITD positivity tends to negatively impact EFS value with Dang (2013) and Ni (2020) have higher value due to its population which only included elderly population. 

Another study that analyzed EFS came with similar results (Yanada et al., 2005; Port et al., 2014). Both studies included patients who received allogeneic stem cell transplantation. Thus, EFS was worse in FLT3-ITD positive patients and has similar risk between transplanted and non-transplanted population.

There are several limitations in this study. First, there is high heterogeneity among the studies included in this meta-analysis, possibly due to the elderly population being the main subject in the study by Dang et al., (2013). Another large cohort study conducted in the elderly population is warranted to confirm our hypothesis. Second, there is heterogeneity among studies in terms of treatment regimen and treatment modality. In several studies, the treatment regimens are standardized for every subject. Meanwhile, there are studies in which the subjects were taken from clinical trials. In these types of studies, there are several treatment regimens that were randomized among the subjects. Moreover, there are 21% of subjects from the study by Niparuck et al., (2019) who only receive best supportive care. The difference between treatment regimen can cause bias in treatment outcome. Third, there are various ways regarding how the study reports OS (e.g., by calculating the HR, by calculating the percentage of OS), thus not all studies could be included in meta-analysis. 

In conclusion, this systematic review and meta-analysis show that FLT3-ITD mutation gives worse prognosis, regardless of whether the population has received transplantation. Thus, it confirms the importance of FLT3-ITD mutation as a prognostic factor in adult, non-transplant patients with AML. Additional gene mutation analyses in patients with AML are needed. As the gene mutation analysis is not appropriate as a routine examination for every patient with AML because of financial issues, clinicians should decide wisely to conduct this examination on particular patients who need it. Furthermore, involving local policy in arranging regulation about FLT3-ITD analysis must become a priority in order to promote its widespread use.
